# Myocardial Creatine Levels Do Not Influence Response to Acute Oxidative Stress in Isolated Perfused Heart

**DOI:** 10.1371/journal.pone.0109021

**Published:** 2014-10-01

**Authors:** Dunja Aksentijević, Sevasti Zervou, Kiterie M. E. Faller, Debra J. McAndrew, Jurgen E. Schneider, Stefan Neubauer, Craig A. Lygate

**Affiliations:** Division of Cardiovascular Medicine, Radcliffe Department of Medicine and British Heart Foundation Centre of Research Excellence, University of Oxford, Oxford, United Kingdom; Instituto Nacional de Cardiologia I. Ch., Mexico

## Abstract

**Background:**

Multiple studies suggest creatine mediates anti-oxidant activity in addition to its established role in cellular energy metabolism. The functional significance for the heart has yet to be established, but antioxidant activity could contribute to the cardioprotective effect of creatine in ischaemia/reperfusion injury.

**Objectives:**

To determine whether intracellular creatine levels influence responses to acute reactive oxygen species (ROS) exposure in the intact beating heart. We hypothesised that mice with elevated creatine due to over-expression of the creatine transporter (CrT-OE) would be relatively protected, while mice with creatine-deficiency (GAMT KO) would fare worse.

**Methods and Results:**

CrT-OE mice were pre-selected for creatine levels 20–100% above wild-type using *in* *vivo* ^1^H^–^MRS. Hearts were perfused in isovolumic Langendorff mode and cardiac function monitored throughout. After 20 min equilibration, hearts were perfused with either H_2_O_2_ 0.5 µM (30 min), or the anti-neoplastic drug doxorubicin 15 µM (100 min). Protein carbonylation, creatine kinase isoenzyme activities and phospho-PKCδ expression were quantified in perfused hearts as markers of oxidative damage and apoptotic signalling. Wild-type hearts responded to ROS challenge with a profound decline in contractile function that was ameliorated by co-administration of catalase or dexrazoxane as positive controls. In contrast, the functional deterioration in CrT-OE and GAMT KO hearts was indistinguishable from wild-type controls, as was the extent of oxidative damage and apoptosis. Exogenous creatine supplementation also failed to protect hearts from doxorubicin-induced dysfunction.

**Conclusions:**

Intracellular creatine levels do not influence the response to acute ROS challenge in the intact beating heart, arguing against creatine exerting (patho-)physiologically relevant anti-oxidant activity.

## Introduction

The canonical role of creatine (Cr) is the transfer and buffering of chemical energy *via* the creatine kinase (CK) reaction, linking ATP production to ATP utilization in cells with high energy demand [1]. However, multiple studies have attributed creatine with both direct and indirect anti-oxidant activity [2]–[6]. For example, there is *in* *vitro* evidence for direct scavenging of free radicals [5], protection of mitochondrial DNA from oxidative damage [4]; and up-regulation of enzymes involved in intracellular oxidative defences [7]. Patients with creatine deficiency syndrome are reported to have increased oxidative stress and ROS-induced apoptotic cell loss [8], while creatine supplementation in senescent mice improved neurobehavioral outcomes and prolonged median survival with a trend towards lower reactive oxygen species (ROS) [9]. More specifically in the heart, our own work has shown that elevating intracellular creatine *via* over-expression of the creatine transporter (CrT) reduces myocardial stunning and ischaemia/reperfusion injury [10]. However, the extent to which anti-oxidant effects may contribute to cardioprotection has not been fully elucidated.

One way to assess this is to challenge intact hearts with known sources of oxidative stress in order to determine whether intracellular creatine levels influence the acute response. For example, hydrogen peroxide (H_2_O_2_) is a relatively stable, membrane diffusible, non-radical species of ROS [11], [12] and the exogenous administration of H_2_O_2_ has been reported to cause a degree of cardiac dysfunction similar to ischemia [13]. Cardiotoxicity predominately arises from the generation of reactive oxygen intermediates such as hydroxyl radicals and singlet oxygen leading to peroxidation of membrane phospholipids, altered membrane permeability, and irreversible loss of cell integrity [14]. Additional mechanisms of toxicity may be related to direct inhibition of glycolytic ATP generation [15], or to redox regulation of regulatory kinases and sarcomeric proteins [12].

A more clinically relevant source of oxidative stress are the antineoplastic anthracycline drugs (e.g. doxorubicin). These are widely used and effective, but dosing is constrained by cardiotoxic side effects which can lead to heart failure [16]–[18]. Doxorubicin is known to accumulate in mitochondrial membranes of cardiomyocytes [17], where a number of complex interactions may occur leading to the generation of superoxide anion, hydrogen peroxide and subsequently hydroxyl radicals *via* a Fenton reaction with iron. The result is oxidation of key proteins with mitochondrial proteins particularly vulnerable, ultimately leading to ATP depletion [16], [18].

Modulating intracellular creatine levels in the heart is not straightforward since cardiomyocytes cannot synthesize creatine [19] and increased dietary creatine does not result in elevated tissue levels [20]. To circumvent this we have previously created mice that constitutively over-express myocardial CrT (CrT-OE) [21]. For the purpose of studying creatine-free hearts, we used mice with knockout of the key biosynthetic enzyme, guanidinoacetate N-methyltransferase (GAMT KO), which have a whole body creatine-deficiency as previously described [22], [23].

Thus the objective of this study was to determine whether the anti-oxidant effects attributed to creatine *in* *vitro* have physiological significance to the intact heart. To address this we measured the effect of acute oxidative stress caused by H_2_O_2_ or doxorubicin on cardiac function in Langendorff perfused mouse hearts. Specifically, we hypothesized that GAMT KO mice with zero creatine in the heart would be more susceptible to ROS-mediated dysfunction, and conversely, that CrT-OE hearts with elevated intracellular creatine would be protected.

## Materials and Methods

### Animal Husbandry

Creatine transporter overexpressing and GAMT KO mice were bred in-house and genotyped as previously described [21]–[23]. Both strains are congenic to C57BL/6J and wild-type littermates (WT) were used as controls. Additional experiments made use of C57BL/6J male mice purchased from Harlan, UK. Mice were housed in specific pathogen-free cages with a 12 h light-dark cycle, controlled humidity and temperature (20–22°C), fed Teklad Global 16% Protein Rodent Diet (naturally creatine-free) and water *ad libitum*. From 6 weeks of age, GAMT KO mice were housed per genotype to prevent creatine ingestion *via* coprophagia. All experiments were approved by institutional ethical review committee and conform to the UK Animals (Scientific Procedures) Act 1986 incorporating European Directive 2010/63/EU.

### *In vivo* ^1^H magnetic resonance spectroscopy (^1^H-MRS)

Myocardial creatine [Cr] was measured non-invasively on a 9.4 T (400 MHz) MR system (Agilent Technologies) using a quadrature-driven birdcage resonator (Rapid Biomedical). CrT- OE mice were anaesthetized with isoflurane and maintained at 1.5–2% in oxygen. Cardiac metabolite signals from a 2 µl voxel, placed in the interventricular septum, were acquired using a double-gated, double spin-echo sequence. Creatine signal was normalized to the water signal and total [Cr] estimated from the average creatine/water ratio using a calibration curve as reported previously [24].

### Heart Perfusions

Mice were anaesthetised by intra-peritoneal injection of pentobarbitone 140 mg/kg. Beating hearts were rapidly excised, cannulated and perfused in the isovolumic Langendorff mode at 80 mmHg perfusion pressure, 37°C with Krebs-Henseleit (KH) buffer continuously gassed with 95% O_2_/5% CO_2_ (pH 7.4) containing (in mM): NaCl 120, KCl 4.7, MgSO_4_.7H_2_O 1.2, NaHCO_3_ 25, KH_2_PO_4_ 1.2, CaCl_2_.H_2_O 1.8, glucose 11. Cardiac function was assessed using a fluid-filled cling film balloon inserted *via* the mitral valve into the left ventricle (LV), and connected *via* a line to a pressure transducer (Memscap) and a Powerlab/8 SP system (AD Instruments). The intraventricular volume was adjusted to achieve an initial LV diastolic pressure of 10–15 mmHg [23]. Functional parameters were averaged for ∼80 cardiac cycles at 5 min perfusion intervals. Left ventricular developed pressure (LVDP) was calculated as the difference between systolic (SP) and diastolic pressures (DP) [25]. Rate pressure product (RPP) was determined as the product of heart rate and LVDP. At the end of each perfusion, hearts were freeze clamped using Wollenberger tongs, cooled in liquid nitrogen and stored at –80°C until further analysis.

### Perfusion protocols

#### Pilot Study 1: H_2_O_2_ dose finding

The effect of H_2_O_2_ on *ex-vivo* function was tested in hearts from C57BL/6J mice at the following concentrations: 200 µM, 100 µM, 50 µM, 5 µM, 1 µM and 0.5 µM (n = 3 for each). Stability of H_2_O_2_ in KH buffer was checked spectrophotometrically using Amplex red hydrogen peroxide/peroxidase assay kit (Invitrogen) and was found to be within 4% and 9% of starting values after 30 min and 60 min perfusion, respectively. H_2_O_2_ KH buffer was therefore always made fresh immediately prior to the perfusion experiment. Perfusion with the H_2_O_2_ scavenger, catalase (150 U/ml, Sigma Aldrich), was used as a positive control (n = 4 per group). Following 20 min equilibration: group 1 was perfused with 0.5 µM H_2_O_2_ for 30 min; group 2 received 20 min pre-treatment with 150 U/ml catalase.

#### Pilot Study 2: Doxorubicin dose finding

Doxorubicin hydrochloride (Dox) (Sigma Aldrich) stock (10 mg/vial) was reconstituted in Milipore-filtered water to a concentration of 10 mM and kept in frozen aliquots away from light until use.

A dose of 15 µM administered over 80 min was chosen from the literature since this has been shown to cause a significant reduction in perfused rat heart function [26]. This dose was confirmed experimentally in perfused C57BL/6J hearts (n = 3) with and without 20 min pre-treatment with the iron-chelator dexrazoxane 300 µM as a positive control (n = 5) (Cardioxane, Novartis).

#### Hydrogen peroxide-induced oxidative stress

After 20 min of functional equilibration, hearts from the following groups were perfused for 30 minutes with 0.5 µM H_2_O_2_ containing KH buffer:

- GAMT KO and WT (n = 4 per group, female, 46 weeks).
- CrT-OE mice pre-selected for moderately elevated cardiac creatine levels (mean 114±7 nmol/mg protein, 72 weeks, n = 7, female) with age-matched WT (mean creatine 65±5 nmol/mg protein, 72 weeks, n = 4, female).

#### Doxorubicin-induced oxidative stress

After 20 min of functional equilibration, hearts from these groups were perfused for 100 minutes with 15 µM Dox containing KH buffer:

- GAMT KO (n = 8, male, 54 weeks) with age-matched WT controls (n = 5, 3 female/2 male).
- CrT-OE mice pre-selected for moderately elevated cardiac creatine levels (mean 114±6 nmol/mg protein, 82 weeks, n = 6 female) with age and sex matched WT controls (creatine 68±5 nmol/mg protein, n = 4).
- C57BL/6J mouse hearts perfused as above (n = 5 male) and with addition of 500 µM creatine monohydrate (Sigma Aldrich) to the perfusate throughout (n = 8, male).

### Biochemical Analysis

ROS induced cardiotoxicity has been linked to the direct oxidative structural and functional modification of all myocardial CK isoforms, with further specific injury of cardiac mitochondrial isoform of CK (MtCK) [27], [28]. Total creatine kinase (Total CK) and individual isoform activities: mitochondrial CK (mito-CK), myofibrilar (MM-CK) and minor cytosolic isoforms (MB-CK and BB-CK) were measured as described before [29].

### Protein Expression Analysis

#### Sample preparation

Snap-frozen hearts were ground and suspended in 350 µl of lysis buffer containing 2% SDS, 50 mM Tris pH 7.5, 150 mmol/l NaCl, 1 mM DTT, protease and phosphatase inhibitor cocktail (cOmplete Ultra EDTA-free and PhoSTOP respectively, Roche). Samples were homogenized using a polytron homogenizer prior to centrifugation at 4°C for 5 minutes at 13,000 rpm. The supernatants were stored at –80°C until protein content was determined by the bicinchoninic acid method (BCA) [30].

#### Carbonylation

20 µg from each sample was used to derivatize to single-strength 2,4 dinitro phenyl hydrazine (DNPH, Camlab Chemicals) in 100% trifluoroacetic acid (TFA, Thermo Scientific) as previously described [31], [32]. The derivatization reaction was stopped by neutralization with 2 M TRIS/30% glycerol. Samples were separated on a pre-cast 12% SDS gel (Thermo Scientific) at 100 V for 90 minutes at room temperature in HEPES buffer (Thermo Scientific) followed by transferring of proteins onto a PVDF membrane (GE Healthcare). Membranes were blocked in 3% BSA in PBS/Tween-20 (0.1%) for 1 hr at room temperature, followed by incubation using a 1° anti-DNPH antibody at 1∶150 (Millipore) for 1 hr at room temperature. DNPH epitopes were detected by a secondary anti-mouse IgG HRP-conjugated antibody (Millipore). Immunoblotted proteins were detected by chemiluminescence using the ECL Advance Kit (GE Healthcare). To confirm equal protein loading, gels were stained using Coomassie blue dye (Sigma Aldrich).

To test the sensitivity of the carbonylation assay, a positive control of oxidised bovine serum albumin was prepared as described in the Millipore OxyBlot manual.

#### p-PKC δ

20 µg of protein extracted in the presence of protease and phosphatase inhibitors was separated on a 12% precast SDS gel (Thermo Scientific). This was followed by a protein transfer to a PVDF membrane, blocking (5% BSA in 1xTBS, 0.01% Tween-20) and an overnight incubation at 4°C in primary antibody (1∶1000) against Thr^505^ phosphorylated PKC δ (Cell Signaling Technologies). HRP-conjugated secondary anti- rabbit IgG antibody (Promega) was incubated at 1∶20,000 for 1 hr at room temperature prior to visualization with the ECL advance chemiluminescence kit (GE Healthcare). As a positive control, MCF-7 breast cancer cells were treated with 0.2 µg/ml Dox for 4 hours in the absence of foetal bovine serum (FBS).

*Quantification* - membranes were stripped and re-probed using an anti α-actinin antibody (Sigma Aldrich) as previously described [30]. Densitometry was performed using a FluoChem 8800 (Alpha Innotech Corporation) and normalised to α actinin.

### Statistical Analysis

The results are presented as mean±SEM. Statistical significance of functional parameters were assessed using Graphpad Prism software v 5.04 by two-way analysis of variance with Bonferroni’s correction for multiple comparisons. For protein markers of oxidative stress, one-way ANOVA with Bonferroni’s correction was used to compare selected groups. Wild-types were compared with genetically-modified mice at baseline, and after Dox and H_2_O_2_ exposure; and exposed hearts were compared to baseline values for the same genotype. Differences were considered significant when P<0.05.

## Results

### Pilot Study 1: H_2_O_2_ dose finding

**Figure 1:**
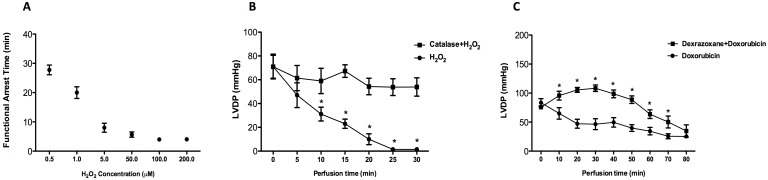
Graded effect of varying doses of H_2_O_2_ in Krebs-Henseleit buffer on functional arrest time (time for LVDP to reduce to 0 mmHg) in isolated perfused mouse hearts (n = 3/group) (A). (**B**) Protective effect of catalase against H_2_O_2_–induced function deterioration. *P<0.01 catalase treated (n = 4) *vs*. H_2_O_2_ treated group (n = 4). (**C**) Protective effect of dexrazoxane on *ex-vivo* cardiac function against doxorubicin (Dox) challenge. *P<0.05 *vs*. dexrazoxane treated hearts (n = 5); Dox (n = 3). LVDP-left ventricular developed pressure. Data mean±S.E.M. P value is for the effect of the genotype by ANOVA with Bonferroni’s correction.

Perfusion of C57BL/6 mouse hearts with H_2_O_2_ (0.5–200 µM) resulted in functional arrest in a dose-dependent manner (Figure 1A), with concentrations >5 µM inducing functional failure and total irreversible cardiac arrest within 5±1 minutes (Figure 1A). A dose of 0.5 µM H_2_O_2_ was therefore chosen for all subsequent perfusions since the deleterious effects were observable over the time course of perfusion, without being too rapid to detect differences in responses between groups. Furthermore, catalase treatment fully inhibited the effect of 0.5 µM H_2_O_2_ on LVDP indicating that positive modulation of experimental outcome was achievable at this dose (Figure 1B).

### Pilot Study 2: Doxorubicin dose finding

A doxorubicin concentration of 15 µM resulted in gradual and significant functional decline (∼70%) during 70 min perfusion. Inclusion of dexrazoxane 300 µM in the perfusion buffer as a positive control significantly delayed the functional decline in response to doxorubicin (Figure 1C).

### Creatine does not protect against acute oxidative stress in the intact heart

There were no significant differences in heart or body weight between CrT-OE and WT controls, while GAMT KO mice exhibited lower heart weight compared to controls, commensurate with a lower body weight as described previously [22] (Table 1). In all experiments, isolated perfused hearts were allowed to equilibrate for 20 min.

**Table 1:** Morphometric parameters of mice used in the study.

	CrT-WT(n = 8F)	CrT-OE(n = 13; 13F)	GAMT WT(n = 9; 7F+2 M)	GAMT KO(n = 12; 4F+8 M)
Age (weeks)	85±3	82±2	50±4	50±4
Body weight (g)	33±3	30±3	27±4	18±1*
Wet heart weight (LV+RV) (mg)	150±10	150±10	200±20	100±10*
Wet heart weight/tibia length (mg/cm)	87±7	86±7	111±10	78±5*
Wet heart weight/body weight (mg/g)	4.6±0.3	4.6±0.2	4.7±0.5	5.8±0.2

LV- left ventricular, RV- right ventricular, All values are mean±SEM.

*P<0.05 versus wild-type for that strain by Student’s t-test.

**Figure 2:**
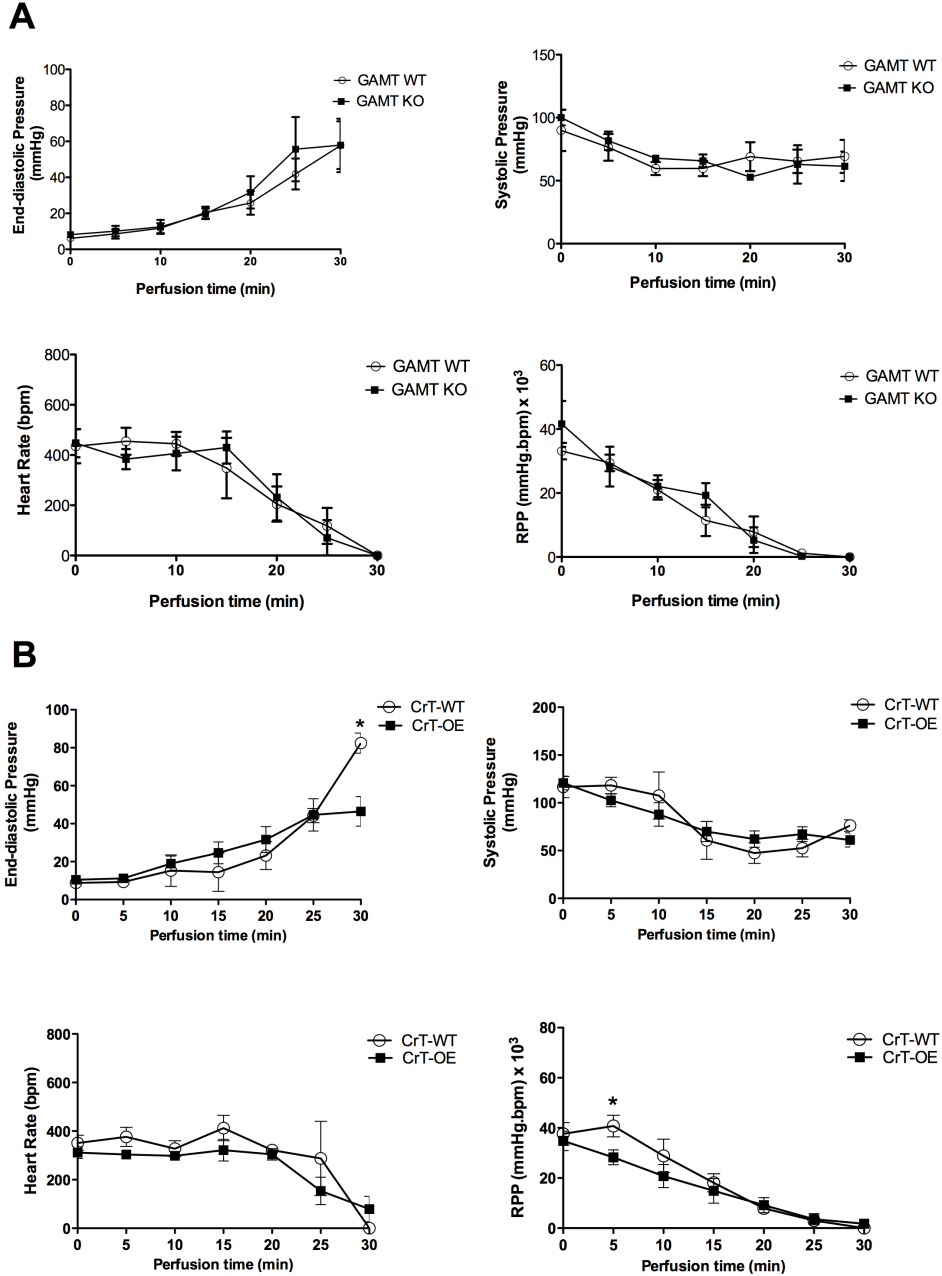
Effect of 0.5 µM H_2_O_2_ on cardiac function during 30 min exposure in isolated perfused GAMT KO hearts and wild-type (WT) controls (A). GAMT KO (n = 4) with the age and sex matched corresponding wild-type (GAMT WT) mice (n = 4; female). (**B**) Effect of 0.5 µM H_2_O_2_ on cardiac function during 30 min exposure in isolated perfused Cr-T overexpressing (CrT-OE) hearts (n = 7; 7 female) and age matched wild-type (CrT-WT) controls (n = 4; female). RPP- rate pressure product. *P<0.05 vs WT. Data mean±S.E.M. P value is for the effect of the genotype by ANOVA with Bonferroni’s correction.

**Figure 3:**
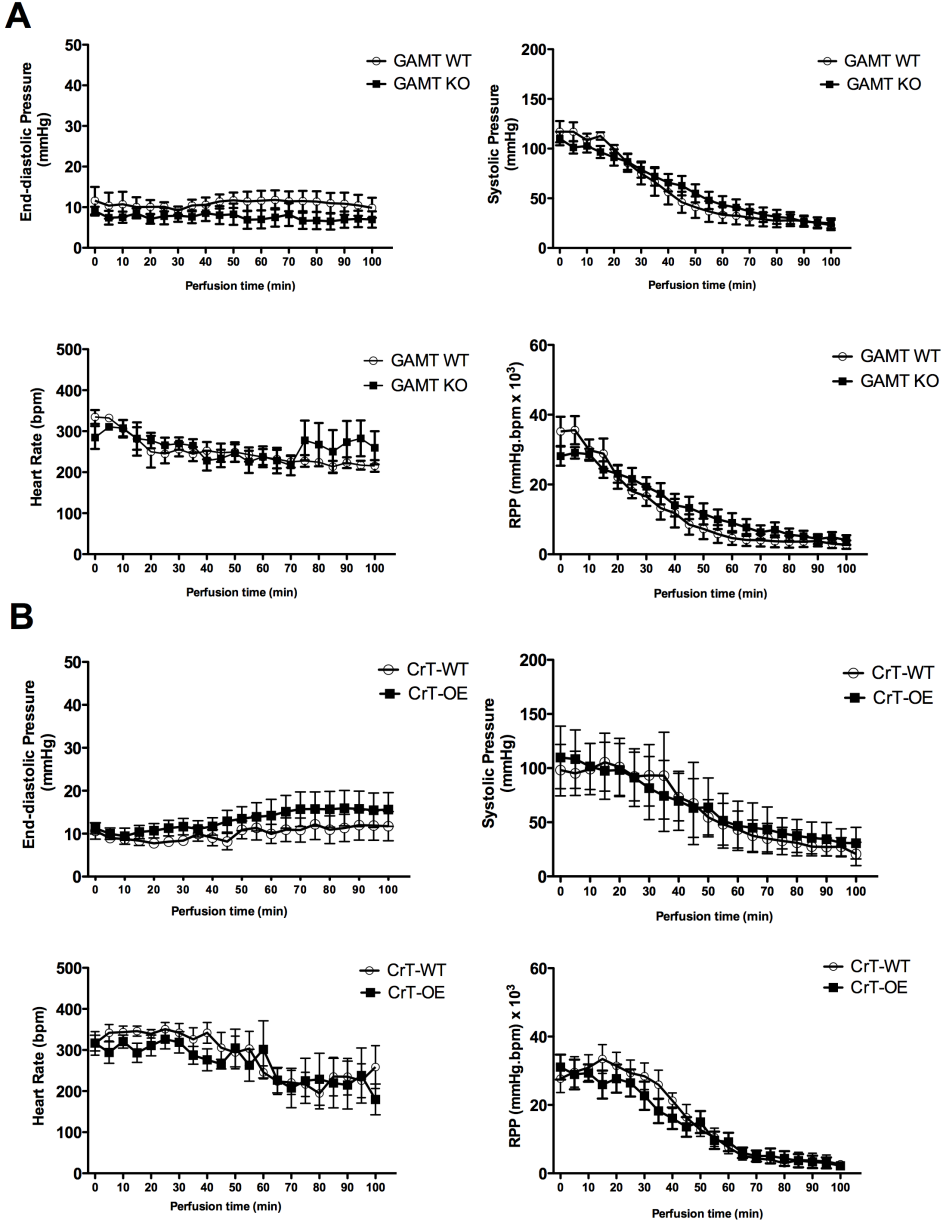
Effect of doxorubicin on cardiac function during 100 min perfusion in: (A) isolated perfused GAMT KO hearts and wild-type (WT) controls. GAMT KO (n = 8; male); WT (n = 5; 3 female+2 male) (**B**) isolated perfused CrT overexpressing hearts (CrT-OE) and wild-type (WT) controls. CrT-OE (n = 6; female); WT (n = 4; female); RPP- rate pressure product. P<0.05 vs WT. Data mean±S.E.M. P value is for the effect of the genotype by ANOVA with Bonferroni’s correction.

There were no significant differences in LV functional parameters prior to oxidative stress challenge in any of our experimental groups when compared to their corresponding WT controls (t = 0 time-point in Figures 2 and 3). Therefore intracellular creatine content had no effect on *ex-vivo* baseline cardiac function.

Perfusion with H_2_O_2_ led to a decline in all LV functional parameters that were virtually indistinguishable between GAMT KO and WT hearts (Figure 2A). A similar response was observed for CrT-OE hearts versus WT controls (Figure 2B), with the exception of significant differences at single-time points for end-diastolic pressure (t = 30 min, Figure 2B) and rate pressure product (t = 5 min, Figure 2B). However, these effects were unsustained and inconsistent, and therefore unlikely to have physiological relevance. Similarly, doxorubicin-induced oxidative stress had comparable effect on cardiac (dys) function of both GAMT KO hearts (Figure 3A) and CrT-OE (Figure 3B) versus their matching WT controls.

**Figure 4:**
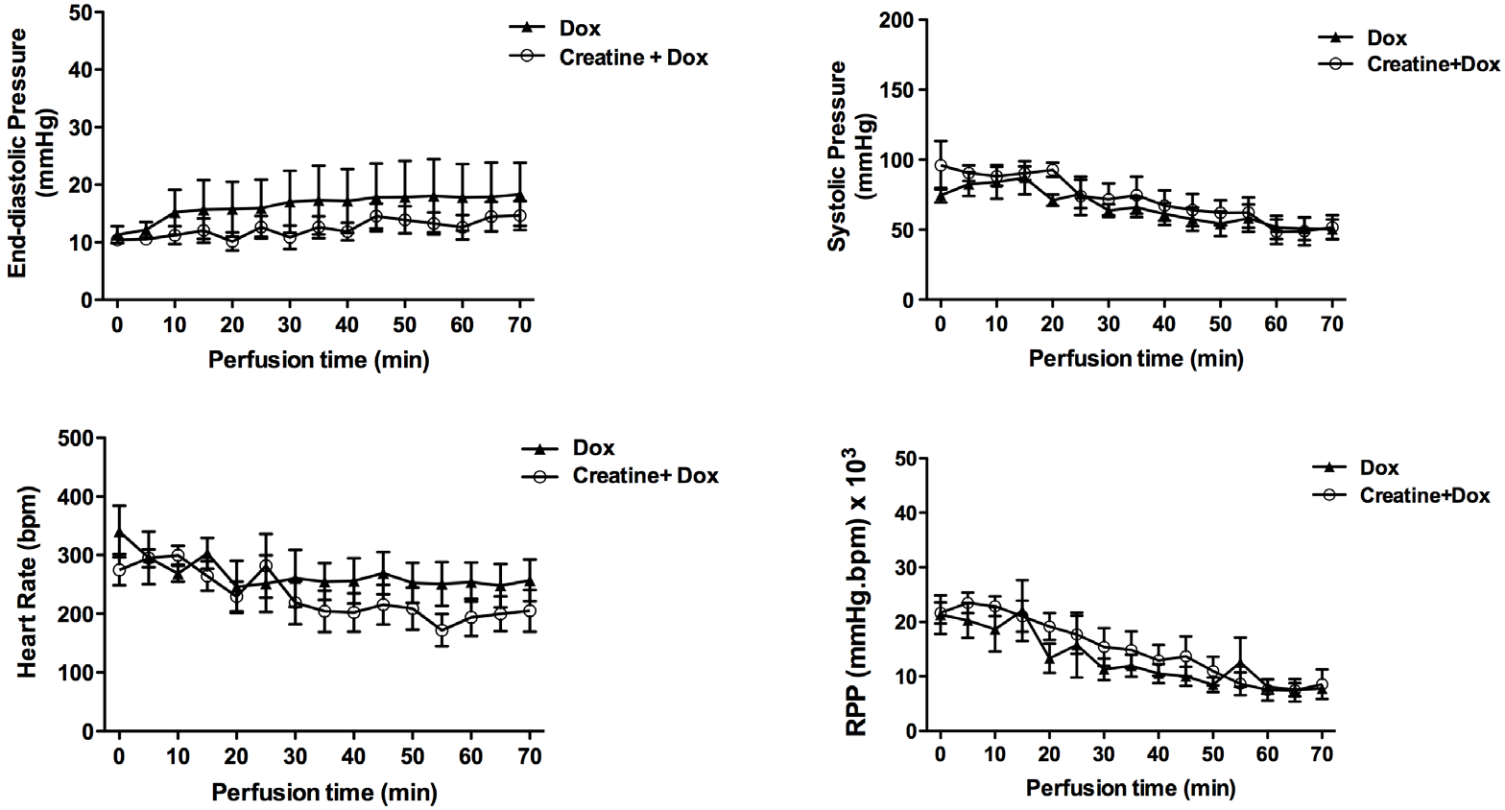
Effect of exogenous creatine supplementation (500 µmol/L) on cardiac function during 70 min doxorubicin (Dox) exposure (n = 8, C57BL/6J; male). RPP- rate pressure product. Data mean±S.E.M.

To determine whether creatine location was important, i.e. extracellular versus intracellular, we administered exogenous creatine monohydrate in the perfusate of doxorubicin-treated C57BL/6 hearts. Exogenous creatine had no effect on baseline *ex-vivo* cardiac function, nor did it ameliorate functional decline compared to doxorubicin-treated hearts alone (Figure 4).

### Markers of oxidative stress and apoptosis

#### Creatine kinase enzyme activity

**Figure 5:**
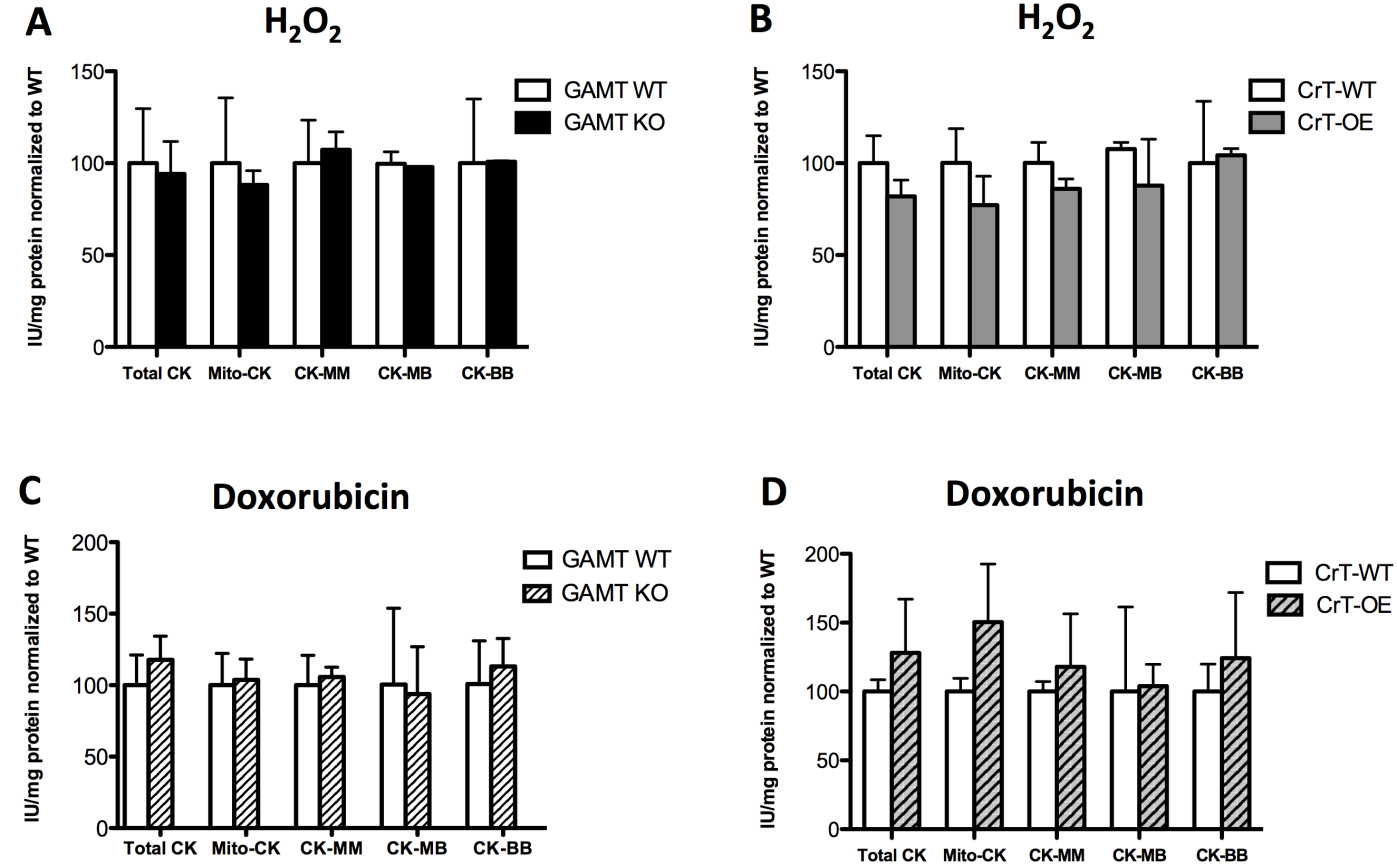
Effect of acute oxidative stress on myocardial creatine kinase (CK) isoform activities: mitochondrial (Mito-CK), myofibrilar (CK-MM), minor cytosolic isoforms (CK-MB, CK-BB): Effect of 0.5 µM H_2_O_2_ on CK isoenzyme activities of (A) GAMT WT (n = 3) and KO (n = 6) (B) CrT-WT (n = 3) and OE (n = 4). Effect of doxorubicin on CK isoenzyme activities of (**C**) GAMT WT (n = 6) and KO (n = 7) and (**D**) CrT-WT (n = 4) and OE (n = 5). Enzyme activities given in IU/mg protein normalized to WT values. Data mean±S.E.M. Comparison with wild-type for that strain by Student’s t-test.

At the cellular level, the activities of total CK and the individual CK isoforms (mitoCK, CK-MM, CK-MB, CK-BB) as markers of intracellular oxidative stress, were not different when compared to their WT controls. A similar response was observed in all genotypes (GAMT KO and CrT-OE) and both oxidative stress agents used (Figure 5).

#### Protein carbonylation

**Figure 6:**
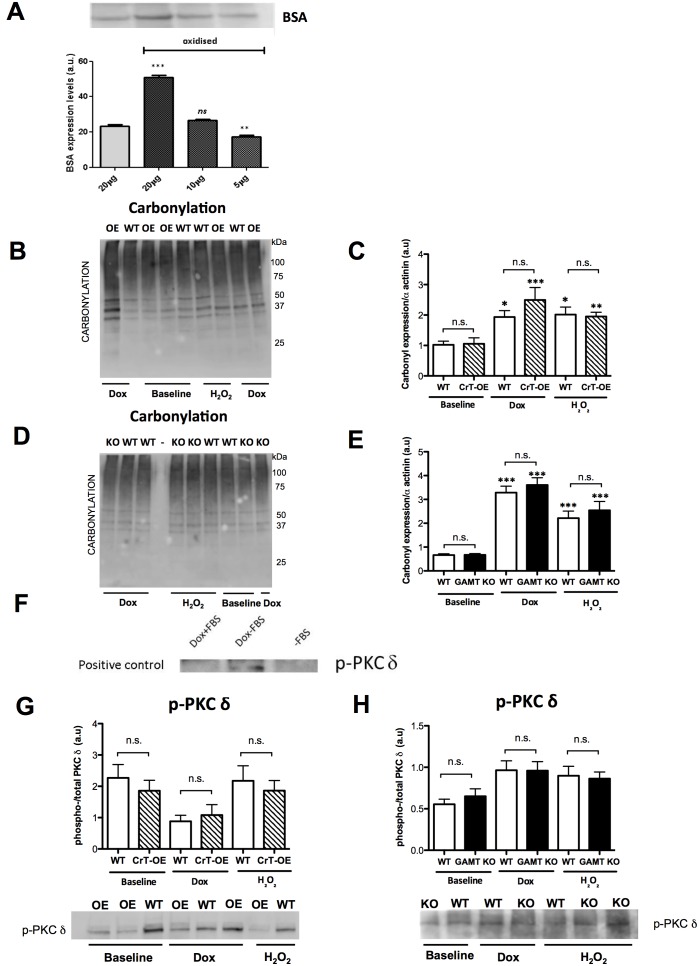
Analysis of phosphorylated PKC δ and carbonylation protein profile in response to acute oxidative stress. (**A**) Oxidized bovine serum albumin (BSA) control demonstrating sensitivity of the assay to detect differences in protein carbonylation. ***P<0.0001 **P = 0.008 *ns*: non-significant. Profile of total carbonylation levels in DNPH-derivatised mouse LV tissue using immunoblotting and probing vs DNPH. Representative blots of (**B**) Wild-type (WT), CrT-OE (OE) and (**D**) GAMT KO (KO) and wild-type (WT). (**C**) Expression of ROS-induced protein damage in lysates of CrT-OE vs WT (**E**) GAMT KO vs WT at baseline, after exposure to doxorubicin hydrochloride (Dox) and H_2_O_2_. (**F**) Representative blot of doxorubicin-treated MCF-7 cells used as positive control displayed increased p-PKC δ levels (Dox–FBS) versus untreated cells (Dox+FBS, –FBS). Phosphorylated PKC δ band was obtained only in the FBS pre-starved cells that were subsequently exposed to Dox. (**G**) Expression of p-PKC δ in CrT-OE vs WT and (**H**) GAMT KO vs WT hearts at baseline, after Dox or H_2_O_2_ treatment [figure includes representative p-PKC δ western blot images for sample groups as above]. Data mean±S.E.M. CrT-OE (n = 5) WT (n = 6) GAMT KO (n = 6) WT (n = 5). *P<0.05, **P<0.01, ***P<0.001 for comparison with baseline values for same genotype by one-way ANOVA with Bonferroni correction.

The ability to detect changes in protein carbonylation was validated by assaying known quantities of non-oxidised and oxidised BSA. Oxidised BSA displayed significantly higher levels of carbonylation when compared to equal amounts (20 µg) of untreated BSA (*P*<0.001; Figure 6A). Levels of protein carbonylation were quantified in mouse heart tissue at baseline and following acute oxidative stress in Langendorff perfused hearts (see Figure 6B, D for representative blots, C–E for quantification). Protein carbonylation was not significantly different at baseline between CrT-OE and WT or GAMT KO and WT, but was elevated in all groups as expected following exposure to either H_2_O_2_ or Dox (Figure 6C & E; One-way ANOVA, *P*<0.001 compared to baseline for both). Crucially, there was no significant difference in the extent of carbonylation between CrT-OE and GAMT KO compared to their respective controls. This confirms on a molecular level that the level of oxidative stress experienced by these hearts was not influenced by intracellular creatine levels.

#### p-PKC δ

The expression of phosphorylated PKC δ was assessed as a marker of apoptotic pathway activation in the same samples as above (representative blot shown in Figure 6G, H). Phospho-PKC δ levels were similar between CrT-OE and WT at baseline and did not significantly change following exposure to either Dox or H_2_O_2_ (One-way ANOVA, *P* = 0.16; Figure 6G). In GAMT KO mice, p-PKC δ was not different at baseline, however, was significantly elevated with exposure to Dox or H_2_O_2_, but to an equal extent for both WT and KO hearts (*P* = 0.0324 versus baseline; Figure 6H). Doxorubicin-treated MCF-7 cells, used as positive control, displayed increased p-PKC δ levels versus untreated (Figure 6F).

## Discussion

This is the first study to examine whether intracellular creatine levels influence the response to acute oxidative stress in the intact beating heart. Here we show that two different ROS sources, H_2_O_2_ and doxorubicin, both cause terminal decline in *ex* *vivo* mouse cardiac function.

This functional deterioration was unaltered in hearts with intracellular creatine ∼75% higher than controls, or in hearts with a complete deficiency of creatine. This was confirmed at the molecular level by measurement of carbonylation, indicating that exposure of proteins to oxidative damage was not influenced by creatine levels. This argues against creatine having significant anti-oxidant activity in the intact heart under acute pathological conditions.

These findings provide insight into our previous report in CrT-OE mice, which showed that similarly elevated levels of myocardial creatine protected against ischaemia/reperfusion injury both *in* *vivo* and *ex* *vivo* [10]. Since I/R injury is associated with a burst of ROS upon reperfusion, then the anti-oxidant effects attributed to creatine could account, in part, for the protective mechanism. Our current work supports the view that the cardio-protective effects of creatine against I/R injury are unrelated to antioxidant activity and are therefore most likely attributable to improved cardiac energetics and protection against mPTP opening as previously described [33]. These protective mechanisms in CrT-OE mice include increased energy reserve in the form of PCr/ATP ratio and glycogen storage, as well as increased energy release from ATP hydrolysis. Since these positive energetic adaptations did not confer protection against acute oxidative stress, suggests that energetic imbalance is not the major cause of dysfunction under these hyper-acute conditions.

One possibility is that the experimental conditions were too harsh to observe a protective effect of creatine in the current study and that subtle effects may have been missed. Certainly, both ROS sources had a rapid and profound effect resulting in irreversible decline and complete cardiac arrest within 30 min for H_2_O_2_ perfusion and 100 min for doxorubicin. Nevertheless, we demonstrated *via* the use of positive controls that this pathology could be beneficially modified. For example, catalase converts two molecules of H_2_O_2_ to O_2_ and H_2_O and was effective in completely protecting against H_2_O_2_ –induced damage.

Similarly, addition of the iron-chelator dexrazoxane altered the time-course of doxorubicin-induced dysfunction, presumably by preventing the formation of hydroxyl radicals *via* the iron-dependent Fenton reaction. It should also be noted that the concentration of 0.5 µM H_2_O_2_ is within the range of estimated exposure levels for cardiomyocytes under pathophysiological conditions, e.g. ischaemia/reperfusion [11], [12]. Likewise, the concentration of doxorubicin we used is similar to the clinically relevant dose observed in plasma of patients after bolus injection [34]. We therefore believe that these are meaningful experimental models and that a robust anti-oxidant effect of creatine would have been readily detectable.

Previous reports have demonstrated cardioprotective effects of exogenously administered phosphocreatine (PCr) in H_2_O_2_ perfused rat hearts [35]. Since creatine is inter-converted to PCr *via* the creatine kinase reaction, it is worth considering why our findings are in apparent disagreement. The key difference is that PCr must exert any protective effect extracellularly, since it is too polar to cross the plasma membrane and is not a substrate for uptake *via* the creatine transporter. That we failed to observe protection from addition of exogenous creatine suggests that the phosphoryl group has functional significance [35]. Exogenous PCr has been shown to directly bind to phospholipid-containing membranes, altering structure and conformation of membrane phospholipids thus protecting membranes from permeabilization by doxorubicin-induced oxidative stress [36], [37].

### Limitations

Although GAMT KO mice are entirely creatine-free, they do accumulate the creatine precursor guanidinoacetate (GA) to relatively high levels, including in the heart. GA can participate in the CK reaction, but the kinetics are insufficient to compensate for the energetic deficit in GAMT KO hearts [23], [38]. However, if GA and creatine both share similar anti-oxidant activity, this could explain why GAMT KO hearts were similarly susceptible to ROS mediated dysfunction.

Nothing is known about the role of GA in myocardial oxidative stress, however, it has previously been shown that GA administration to rat striatum reduces antioxidant capacity [39], arguing against a compensatory protective role from GA accumulation.

In our study we used mature mice (≥1 year) which are more representative of the age of patients with heart disease [40], patients with anthracycline induced cardiomyopathy [41] and in line with current recommendations to include older mice in pre-clinical studies [42]. However, for pragmatic reasons this necessitated the use of both sexes, and we were careful to age- and sex- match groups throughout the study. Unfortunately, in one experimental group (doxorubicin-treated GAMT WT) we had insufficient mice of the appropriate genotype >1 year old and therefore had to use mixed gender (n = 3 females n = 2 males) rather than an all male group. Cleary this is non-ideal, however, we firmly believe it is highly unlikely to affect the outcome of the study for a number of reasons. Firstly, there was no divergence in response when individual traces were plotted together. Furthermore, in contrast to other mammalian species, female mice have age-associated mitochondrial superoxide generation comparable to males [43]. In addition, although estrogen can protect female mice from ischaemia/reperfusion injury [44], our three female mice are unlikely to benefit from estrogen-mediated cardioprotection since estrogen levels decline by 75% between 6 and 18 months [45]. Lastly, our study used retrograde *ex-vivo* Langendorff perfusion with crystalloid Krebs-Henseleit buffer and is therefore lacking in circulating hormones that may exert gender specific cardioprotection *in-vivo*.

Specifically, in our study we do not rule out that creatine may have a protective role against the gradual functional decline observed in chronic *in* *vivo* models of doxorubicin toxicity. There is a large body of evidence describing impaired cardiac energetics in response to doxorubicin dosing, including decreased PCr and ATP levels, inactivation of mitochondrial respiratory enzymes and altered substrate utilisation (see [46] for comprehensive review).

Recent findings also show reduced creatine kinase (CK) activity [47] and impaired creatine uptake capacity due to down-regulation of the creatine transporter [48]. Most notably, genetic over-expression of the muscle isoform of CK in heart improved cardiac function and survival in a chronic mouse model of doxorubicin toxicity [49]. A reduction in plasma markers of cytotoxicity has also been observed with oral creatine supplementation in rats, although the effect on cardiac function was not reported [50]. Clearly, it remains possible that increasing intracellular creatine levels might protect against anthracycline toxicity in long-term experiments *via* energetic mechanisms.

## Conclusion

Using two genetic models of chronically altered myocardial creatine (supra-physiological elevation and zero-creatine) we have demonstrated that intracellular creatine content does not influence the detrimental effects of acute oxidative stress on cardiac function. This argues against creatine possessing significant antioxidant activity under acute pathological conditions in the intact beating heart.

## References

[1] Neubauer S (2007) The failing heart–an engine out of fuel. New Eng J Med 356: 1140–1151. 17360992 10.1056/NEJMra063052

[2] Berneburg M, Gremmel T, Kurten V, Schroeder P, Hertel I, et al (2005) Creatine supplementation normalizes mutagenesis of mitochondrial DNA as well as functional consequences. J Invest Dermatol 125: 213–220. 16098029 10.1111/j.0022-202X.2005.23806.x

[3] Fimognari C, Sestili P, Lenzi M, Cantelli-Forti G, Hrelia P (2009) Protective effect of creatine against RNA damage. Mutat Res 670: 59–67. 19631670 10.1016/j.mrfmmm.2009.07.005

[4] Guidi C, Potenza L, Sestili P, Martinelli C, Guescini M, et al (2008) Differential effect of creatine on oxidatively-injured mitochondrial and nuclear DNA. Biochim Biophys Acta 1780: 16–26. 18022765 10.1016/j.bbagen.2007.09.018

[5] Lawler JM, Barnes WS, Wu G, Song W, Demaree S (2002) Direct antioxidant properties of creatine. Biochem Biophys Res Commun 290: 47–52. 11779131 10.1006/bbrc.2001.6164

[6] Sestili P, Martinelli C, Bravi G, Piccoli G, Curci R, et al (2006) Creatine supplementation affords cytoprotection in oxidatively injured cultured mammalian cells via direct antioxidant activity. Free Radic Biol Med 40: 837–849. 16520236 10.1016/j.freeradbiomed.2005.10.035

[7] Young JF, Larsen LB, Malmendal A, Nielsen NC, Straadt IK, et al (2010) Creatine-induced activation of antioxidative defence in myotube cultures revealed by explorative NMR-based metabonomics and proteomics. J Int Soc Sports Nutr 7: 9. 20205771 10.1186/1550-2783-7-9 PMC2822831

[8] Alcaide P, Merinero B, Ruiz-Sala P, Richard E, Navarrete R, et al (2011) Defining the pathogenicity of creatine deficiency syndrome. Hum Mutat 32: 282–291. 21140503 10.1002/humu.21421

[9] Bender A, Beckers J, Schneider I, Holter SM, Haack T, et al (2008) Creatine improves health and survival of mice. Neurobiol Aging 29: 1404–1411. 17416441 10.1016/j.neurobiolaging.2007.03.001

[10] Lygate CA, Bohl S, ten Hove M, Faller KM, Ostrowski PJ, et al (2012) Moderate elevation of intracellular creatine by targeting the creatine transporter protects mice from acute myocardial infarction. Cardiovasc Res 96: 466–475. 22915766 10.1093/cvr/cvs272 PMC3500046

[11] Schroder E, Eaton P (2008) Hydrogen peroxide as an endogenous mediator and exogenous tool in cardiovascular research: issues and considerations. Curr Opin Pharmacol 8: 153–159. 18243791 10.1016/j.coph.2007.12.012

[12] Avner BS, Hinken AC, Yuan C, Solaro RJ (2010) H2O2 alters rat cardiac sarcomere function and protein phosphorylation through redox signaling. Am J Physiol Heart Circ Physiol 299: H723–730. 20562337 10.1152/ajpheart.00050.2010 PMC2944474

[13] Canton M, Neverova I, Menabo R, Van Eyk J, Di Lisa F (2004) Evidence of myofibrillar protein oxidation induced by postischemic reperfusion in isolated rat hearts. Am J Physiol Heart Circ Physiol 286: H870–877. 14766672 10.1152/ajpheart.00714.2003

[14] Janero DR, Hreniuk D, Sharif HM (1991) Hydrogen peroxide-induced oxidative stress to the mammalian heart-muscle cell (cardiomyocyte): lethal peroxidative membrane injury. J Cell Physiol 149: 347–364. 1744169 10.1002/jcp.1041490302

[15] Chatham JC, Gilbert HF, Radda GK (1989) The metabolic consequences of hydroperoxide perfusion on the isolated rat heart. Eur J Biochem 184: 657–662. 2806248 10.1111/j.1432-1033.1989.tb15063.x

[16] Maslov MY, Chacko VP, Hirsch GA, Akki A, Leppo MK, et al (2010) Reduced in vivo high-energy phosphates precede adriamycin-induced cardiac dysfunction. Am J Physiol Heart Circ Physiol 299: H332–337. 20495142 10.1152/ajpheart.00727.2009 PMC2930382

[17] Tokarska-Schlattner M, Wallimann T, Schlattner U (2006) Alterations in myocardial energy metabolism induced by the anti-cancer drug doxorubicin. C R Biol 329: 657–668. 16945832 10.1016/j.crvi.2005.08.007

[18] Gratia S, Kay L, Potenza L, Seffouh A, Novel-Chate V, et al (2012) Inhibition of AMPK signalling by doxorubicin: at the crossroads of the cardiac responses to energetic, oxidative, and genotoxic stress. Cardiovasc Res 95: 290–299. 22461523 10.1093/cvr/cvs134

[19] Snow RJ, Murphy RM (2001) Creatine and the creatine transporter: a review. Mol Cell Biochem 224: 169–181. 11693194 10.1023/a:1011908606819

[20] Boehm E, Chan S, Monfared M, Wallimann T, Clarke K, et al (2003) Creatine transporter activity and content in the rat heart supplemented by and depleted of creatine. Am J Physiol Endocrinol Metab 284: E399–406. 12531746 10.1152/ajpendo.00259.2002

[21] Wallis J, Lygate CA, Fischer A, ten Hove M, Schneider JE, et al (2005) Supranormal myocardial creatine and phosphocreatine concentrations lead to cardiac hypertrophy and heart failure: insights from creatine transporter-overexpressing transgenic mice. Circulation 112: 3131–3139. 16286605 10.1161/CIRCULATIONAHA.105.572990

[22] Schmidt A, Marescau B, Boehm EA, Renema WK, Peco R, et al (2004) Severely altered guanidino compound levels, disturbed body weight homeostasis and impaired fertility in a mouse model of guanidinoacetate N-methyltransferase (GAMT) deficiency. Hum Mol Genet 13: 905–921. 15028668 10.1093/hmg/ddh112

[23] ten Hove M, Lygate CA, Fischer A, Schneider JE, Sang AE, et al (2005) Reduced inotropic reserve and increased susceptibility to cardiac ischemia/reperfusion injury in phosphocreatine-deficient guanidinoacetate-N-methyltransferase-knockout mice. Circulation 111: 2477–2485. 15883212 10.1161/01.CIR.0000165147.99592.01

[24] Schneider JE, Tyler DJ, ten Hove M, Sang AE, Cassidy PJ, et al (2004) In vivo cardiac 1H-MRS in the mouse. Magn Reson Med 52: 1029–1035. 15508174 10.1002/mrm.20257

[25] Ogino H, Smolenski RT, Zych M, Seymour AM, Yacoub MH (1996) Influence of preconditioning on rat heart subjected to prolonged cardioplegic arrest. Ann Thorac Surg 62: 469–474. 8694607

[26] Pelikan PC, Weisfeldt ML, Jacobus WE, Miceli MV, Bulkley BH, et al (1986) Acute doxorubicin cardiotoxicity: functional, metabolic, and morphologic alterations in the isolated, perfused rat heart. J Cardiovasc Pharmacol 8: 1058–1066. 2429080

[27] Tokarska-Schlattner M, Wallimann T, Schlattner U (2002) Multiple interference of anthracyclines with mitochondrial creatine kinases: preferential damage of the cardiac isoenzyme and its implications for drug cardiotoxicity. Mol Pharmacol 61: 516–523. 11854431 10.1124/mol.61.3.516

[28] Muller M, Moser R, Cheneval D, Carafoli E (1985) Cardiolipin is the membrane receptor for mitochondrial creatine phosphokinase. J Biol Chem 260: 3839–3843. 3972849

[29] Lygate CA, Hunyor I, Medway D, de Bono JP, Dawson D, et al (2009) Cardiac phenotype of mitochondrial creatine kinase knockout mice is modified on a pure C57BL/6 genetic background. J Mol Cell Cardiol 46: 93–99. 18948110 10.1016/j.yjmcc.2008.09.710

[30] ten Hove M, Makinen K, Sebag-Montefiore L, Hunyor I, Fischer A, et al (2008) Creatine uptake in mouse hearts with genetically altered creatine levels. J Mol Cell Cardiol 45: 453–459. 18602925 10.1016/j.yjmcc.2008.05.023 PMC2568826

[31] Divald A, Kivity S, Wang P, Hochhauser E, Roberts B, et al (2010) Myocardial ischemic preconditioning preserves postischemic function of the 26S proteasome through diminished oxidative damage to 19S regulatory particle subunits. Circ Res 106: 1829–1838. 20431057 10.1161/CIRCRESAHA.110.219485

[32] Wang P, Powell SR (2010) Decreased sensitivity associated with an altered formulation of a commercially available kit for detection of protein carbonyls. Free Radic Biol Med 49: 119–121. 20230891 10.1016/j.freeradbiomed.2010.03.005 PMC2896977

[33] Lygate CA, Bohl S, ten Hove M, Faller KM, Ostrowski PJ, et al (2012) Moderate elevation of intracellular creatine by targeting the creatine transporter protects mice from acute myocardial infarction. Cardiovasc Res 96: 466–475. 22915766 10.1093/cvr/cvs272 PMC3500046

[34] Tokarska-Schlattner M, Zaugg M, Zuppinger C, Wallimann T, Schlattner U (2006) New insights into doxorubicin-induced cardiotoxicity: the critical role of cellular energetics. J Mol Cell Cardiol 41: 389–405. 16879835 10.1016/j.yjmcc.2006.06.009

[35] Zucchi R, Poddighe R, Limbruno U, Mariani M, Ronca-Testoni S, et al (1989) Protection of isolated rat heart from oxidative stress by exogenous creatine phosphate. J Mol Cell Cardiol 21: 67–73. 10.1016/0022-2828(89)91494-6 2716067

[36] Anyukhovsky EP, Javadov SA, Preobrazhensky AN, Beloshapko GG, Rosenshtraukh LV, et al (1986) Effect of phosphocreatine and related compounds on the phospholipid metabolism of ischemic heart. Biochem Med Metab Biol 35: 327–334. 3718764 10.1016/0885-4505(86)90090-3

[37] Tokarska-Schlattner M, Epand RF, Meiler F, Zandomeneghi G, Neumann D, et al (2012) Phosphocreatine interacts with phospholipids, affects membrane properties and exerts membrane-protective effects. PLOS ONE 7: e43178. 22912820 10.1371/journal.pone.0043178 PMC3422282

[38] Lygate CA, Aksentijevic D, Dawson D, ten Hove M, Phillips D, et al (2013) Living without creatine: unchanged exercise capacity and response to chronic myocardial infarction in creatine-deficient mice. Circ Res 112: 945–955. 23325497 10.1161/CIRCRESAHA.112.300725 PMC4182017

[39] Zugno AI, Stefanello FM, Scherer EB, Mattos C, Pederzolli CD, et al (2008) Guanidinoacetate decreases antioxidant defenses and total protein sulfhydryl content in striatum of rats. Neurochem Res 33: 1804–1810. 18343996 10.1007/s11064-008-9636-6

[40] NICE (2010) Management of chronic heart failure in adults in primary and secondary care http://www.nice.org.uk/guidance/cg108.

[41] Cardinale D, Colombo A, Lamantia G, Colombo N, Civelli M, et al (2010) Anthracycline-induced cardiomyopathy: clinical relevance and response to pharmacologic therapy. J Am Coll Cardiol 55: 213–220. 20117401 10.1016/j.jacc.2009.03.095

[42] Hausenloy DJ, Baxter G, Bell R, Botker HE, Davidson SM, et al (2010) Translating novel strategies for cardioprotection: the Hatter Workshop Recommendations. Basic Res Cardiol 105: 677–686. 20865418 10.1007/s00395-010-0121-4 PMC2965360

[43] Ali SS, Xiong C, Lucero J, Behrens MM, Dugan LL, et al (2006) Gender differences in free radical homeostasis during aging: shorter-lived female C57BL6 mice have increased oxidative stress. Aging Cell 5: 565–574. 17129217 10.1111/j.1474-9726.2006.00252.x

[44] Deschamps AM, Murphy E, Sun J (2010) Estrogen receptor activation and cardioprotection in ischemia reperfusion injury. Trends Cardiovas Med 20: 73–78. 10.1016/j.tcm.2010.05.001 PMC3011947 21130949

[45] Sun LY, Wang N, Ban T, Sun YH, Han Y, et al (2014) MicroRNA-23a mediates mitochondrial compromise in estrogen deficiency-induced concentric remodeling via targeting PGC-1alpha. J Mol Cell Cardiol 75C: 1–11. 10.1016/j.yjmcc.2014.06.012 24984145

[46] Tokarska-Schlattner M, Wallimann T, Schlattner U (2006) Alterations in myocardial energy metabolism induced by the anti-cancer drug doxorubicin. C R Biol 329: 657–668. 16945832 10.1016/j.crvi.2005.08.007

[47] Streijger F, Pluk H, Oerlemans F, Beckers G, Bianco AC, et al (2009) Mice lacking brain-type creatine kinase activity show defective thermoregulation. Physiol Behav 97: 76–86. 19419668 10.1016/j.physbeh.2009.02.003 PMC3133955

[48] Darrabie MD, Arciniegas AJL, Mantilla JG, Mishra R, Vera MP, et al (2012) Exposing cardiomyocytes to subclinical concentrations of doxorubicin rapidly reduces their creatine transport. Am J Physiol Heart Circ Physiol 303: H539–H548. 22752631 10.1152/ajpheart.00108.2012

[49] Gupta A, Rohlfsen C, Leppo MK, Chacko VP, Wang Y, et al (2013) Creatine kinase-overexpression improves myocardial energetics, contractile dysfunction and survival in murine doxorubicin cardiotoxicity. PLOS ONE 8: e74675. 24098344 10.1371/journal.pone.0074675 PMC3788056

[50] Santos RV, Batista ML Jr, Caperuto EC, Costa Rosa LF (2007) Chronic supplementation of creatine and vitamins C and E increases survival and improves biochemical parameters after Doxorubicin treatment in rats. Clin Exp Pharmacol Physiol 34: 1294–1299. 17973871 10.1111/j.1440-1681.2007.04717.x

